# Post-traumatic stress disorder in Australia: Results from the 2020–2022 National Study of Mental Health and Wellbeing

**DOI:** 10.1177/00048674261450374

**Published:** 2026-06-16

**Authors:** Cynthia M Kroeger, Sam Jones, Amy-Leigh Rowe, Lucinda Grummitt, Siobhan M O’Dean, Kirsty Rowlinson, Emma L Barrett, Cath Chapman, Katherine L Mills

**Affiliations:** The Matilda Centre for Research in Mental Health and Substance Use, The University of Sydney, Sydney, NSW, Australia

**Keywords:** Post-traumatic stress disorder, lifetime prevalence, remission, population mental health, sex differences

## Abstract

**Background:**

Post-traumatic stress disorder is a leading driver of distress and premature mortality.

**Objective:**

To provide contemporary, nationally representative estimates of post-traumatic stress disorder in Australia using data from the Australian population-based 2020–2022 National Study of Mental Health and Wellbeing, including sex-specific prevalence, remission and comparisons with the 2007 National Study of Mental Health and Wellbeing.

**Methods:**

Post-traumatic stress disorder and 14 other *Diagnostic and Statistical Manual of Mental Disorders* (4th ed.) disorders were assessed with the World Mental Health Composite International Diagnostic Interview 3.0. Kaplan–Meier survival analysis estimated projected lifetime risk and time to remission. Cox regression tested for projected lifetime remission. Logistic regression tested associations between post-traumatic stress disorder and demographic, mental health, and physical health variables, and prevalence changes between the 2007 and 2020–2022 surveys.

**Results:**

Lifetime post-traumatic stress disorder prevalence (*n* = 15,893) was 7.1%. Females had higher odds than males (odds ratio = 2.3, confidence interval = [2.0, 2.7]) of post-traumatic stress disorder diagnosis. Projected lifetime risk was 9.6% and higher for females (12.3%) than males (6.4%; hazard ratio = 2.2, standard error = 0.10). Projected lifetime remission was 63.3%, with higher remission for males than females (68.7% vs 61.2%; hazard ratio = 0.81; standard error = 0.10). Three quarters entered remission after 10 years for males and 20 years for females. Individuals with post-traumatic stress disorder had higher odds of experiencing any anxiety disorder (adjusted odds ratio = 6.0, confidence interval = [5.0, 7.3]), any mood disorder (adjusted odds ratio = 4.8, confidence interval = [3.9, 5.9]), sedative use disorder (adjusted odds ratio = 7.9, confidence interval = [3.9, 15.9]), suicidality (adjusted odds ratio = 5.0, confidence interval = [4.2, 5.9]), asthma (adjusted odds ratio = 2.0, confidence interval = [1.7, 2.4]) and diabetes (adjusted odds ratio = 1.8, confidence interval = [1.2, 2.6]). No differences in post-traumatic stress disorder diagnosis were observed between the 2007 and 2020–2022 surveys.

**Conclusion:**

Post-traumatic stress disorder remains a prevalent and chronic condition in Australia, frequently co-occurring with other mental and physical health conditions and characterised by significant sex differences in risk and remission.

## Introduction

Post-traumatic stress disorder (PTSD) is a mental health condition that can arise following exposure to traumatic events such as combat, physical and sexual assault, natural disasters, or serious accidents. Although the diagnostic criteria for PTSD has changed over time, at its core, PTSD is characterised by symptoms of intrusion or re-experiencing, avoidance and heightened arousal (APA, 2022). While many individuals recover, a substantial proportion experience chronic or recurring symptoms (Santiago et al., 2013). A previous national survey in Australia estimated that only 14.9% of people with PTSD will remit within 12 months of symptom onset (Chapman et al., 2012). Internationally, systematic reviews of epidemiological studies have shown that around half of PTSD cases persist beyond 3–7 years (Morina et al., 2014; Steinert et al., 2015). Consistently, PTSD is a leading contributor to psychological distress, functional impairment and economic burden (Amedu and Dwarika, 2024; Jellestad et al., 2021).

PTSD is also associated with substantial functional impairment and elevated risk for chronic illness, suicidality and premature mortality (Nilaweera et al., 2023; Schlenger et al., 2015). A recent meta-analysis of 34 studies across 9 countries found that individuals with PTSD experience significant impairments across most functional domains compared to those without PTSD (Jellestad et al., 2021). Females consistently show higher prevalence and report more severe acute stress responses than males, potentially driving disparities in symptom burden and remission (Darves-Bornoz et al., 2008; Luz et al., 2016). PTSD frequently co-occurs with depression, substance use, chronic pain, cardiovascular disease and metabolic disorders (Brennstuhl et al., 2015; Edmondson and von Kanel, 2017; Shalev et al., 2017),

Despite this public health significance, substantial gaps remain in our epidemiological understanding of PTSD. Few large-scale studies have been conducted since the original World Mental Health Surveys (2001–2012; Kessler et al., 2022). In Australia, the recent nationally representative prevalence data come from the 2007 National Survey of Mental Health and Wellbeing (NSMHWB), now more than 15 years old (Chapman et al., 2012). The 2007 NSMHWB estimated a lifetime PTSD prevalence of 7.2% and projected that although most individuals with PTSD would eventually remit (approximately 92%), the median time to remission was 14 years. Internationally, lifetime PTSD prevalence varies widely depending on methods and setting, with estimates ranging from 0.4% in China (Zhou et al., 2021) to 8.8% in Northern Ireland (Ferry et al., 2014). Since then, limited research has examined PTSD remission patterns, sex-specific trajectories, or co-occurring mental and physical health profiles using updated population data. Few studies have incorporated projection modelling, sex-disaggregated remission estimates, temporal sequencing of PTSD with co-occurring mental health conditions or consistent diagnostic methods across time, limiting their utility for trend analysis and health services planning.

This study addresses these evidence gaps by drawing on data from the 2020–2022 NSMHWB to provide a current, nationally representative picture of PTSD in Australia. Specifically, it aims to: (1) estimate the sex-specific prevalence, projected prevalence, and projected lifetime remission rates of PTSD in the Australian population; (2) compare current PTSD prevalence estimates to those from the 2007 National Survey; (3) examine associations between PTSD and a comprehensive range of demographic characteristics, mental health conditions and physical health conditions; and (4) determine the order of onset of PTSD with co-occurring mental health conditions. By producing a robust update on the burden of PTSD, this study contributes essential evidence to guide public mental health strategy, resource planning and approaches to care in Australia.

## Methods

### Sample

This study used data from the 2020–2022 NSMHWB, an Australian Government-funded survey conducted by the Australian Bureau of Statistics (ABS, 2023). The NSMHWB is a nationally representative survey of Australians aged 16–85 years living in private dwellings across all states and territories. People living in very remote areas and discrete Aboriginal and Torres Strait Islander communities were excluded. A total of 15,893 individuals participated across two collection waves (2020–2021 and 2021–2022), selected through stratified area sampling. Households were randomly selected, with one respondent per household randomly chosen. The overall response rate was 52%. Survey weights were applied to reflect a population of 19.8 million adults.

For historical comparison, this study also includes data from the 2007 NSMHWB (*n* = 8841; 60% response rate). Both surveys used consistent methods and instruments for the assessment of PTSD, allowing for valid longitudinal analysis (Chapman et al., 2012; Sunderland et al., 2024). As this is a secondary analysis of anonymised, publicly available ABS data, ethics approval was not required.

### Measures

All data were collected using structured interviews conducted primarily in person, with video conferencing used during COVID-19 lockdown periods.

#### PTSD assessment

PTSD and other mental disorders were assessed using a modified version of the World Mental Health Organization Composite International Diagnostic Interview, version 3.0 (WMH-CIDI; Kessler and Ustün, 2004), a fully structured lay-administered interview developed for use in large population surveys and implemented internationally through the World Mental Health Survey Initiative (Scott et al., 2018). The WMH-CIDI generates diagnoses based on algorithms that operationalise *Diagnostic and Statistical Manual of Mental Disorders* (4th ed.; DSM-IV) criteria. These represent standardised survey-based diagnoses rather than clinician-confirmed clinical diagnoses.

In both the 2007 and 2020–2022 surveys, the PTSD module began by assessing lifetime exposure to 29 potentially traumatic events (PTEs; Kroeger et al., 2025; Mills et al., 2011). Respondents who reported at least one PTE were asked whether the event led to psychological distress (e.g. intrusive memories, emotional numbness, concentration difficulties, hyperarousal). Only those who endorsed such symptoms were administered the full PTSD module. In cases of multiple PTEs, respondents identified a ‘worst event’, to which all subsequent PTSD questions referred.

#### Remission

Participants were asked to report the age at which they first experienced symptoms of re-experiencing, avoidance or hyperarousal, along with how long each symptom cluster persisted. The longest duration was added to the age of onset to estimate the age at which PTSD symptoms most recently occurred. Individuals were classified as remitted if their most recent episode ended more than 12 months before the time of the interview. These methods are consistent with those used previously (Chapman et al., 2012).

#### Demographic data

Cisgender identity was assessed by comparing sex assigned at birth with current gender identity. Participants were classified as cisgender if their gender identity aligned with their sex assigned at birth (male or female). Remoteness of residence was derived from the Accessibility/Remoteness Index of Australia (ABS, 2016). Socioeconomic disadvantage was derived from the 2021 ABS Index of Relative Socio-economic Disadvantage, a component of the Socio-Economic Indexes for Areas (ABS, 2021).

#### Mental health conditions

For individuals who met the criteria for both PTSD and another disorder, age of onset data were used to determine whether PTSD was temporally primary, occurred in the same year (simultaneous) or was temporally secondary, as done previously (Chapman et al., 2012). Analyses were conducted at the level of disorder groups – namely anxiety, mood and substance use disorders – using the earliest reported onset within each group.

#### Suicide and self-harm

Suicidal thoughts, behaviours and self-harm were assessed. In the 2020–2022 survey, self-harm (intentional self-injury without suicidal intent) was assessed for the first time using the question: ‘Have you ever intentionally harmed yourself in any way, but not with the intention of taking your life?’ Examples also were provided.

#### Physical health conditions

Participants were asked if they had been told by a doctor or nurse that they currently had any long-term health conditions expected to last 6 months or more. Conditions examined in this study include arthritis, osteoporosis, asthma, cancer, diabetes, heart disease, stroke, kidney disease and bronchitis or emphysema.

### Analyses

All statistical analyses were performed in R (version 4.4.1) within the secure ABS DataLab environment. Prevalence estimates were generated using replicate weights provided by the ABS to conform with independent population estimates. Standard errors were calculated using the delete-a-group jackknife variance technique. Estimates of projected lifetime risk, age at selected age-of-onset percentiles and median time to remission were generated using Kaplan–Meier survival estimates. Tied survival times were handled using the Efron approximation of the partial risk and all variables were screened for proportionality of hazards and outliers (Grambsch and Therneau, 1994). Projected lifetime remission rates were generated using Cox proportional hazards models, with sex at birth compared using a hazard ratio (HR) with 95% confidence interval. To examine changes in PTSD prevalence over time, data from the 2007 and 2020–2022 surveys were pooled to a common dataset (Moser et al., 2013). Logistic regression models were specified with survey year as the independent variable (coded 0 for 2007 and 1 for 2020–2022) to test for statistically significant differences in prevalence across time. Associations between PTSD and demographic, mental and physical health variables were evaluated using logistic regression models, with odds ratios (ORs) and corresponding 95% confidence intervals (CIs) reported. Individual contributions of variables were assessed using adjusted Wald *F* statistics.

## Results

### Lifetime and 12-month prevalence of PTSD

Among Australian adults, 7% met the criteria for a lifetime diagnosis of PTSD and 4% were symptomatic in the past 12 months. Lifetime diagnosis of PTSD accounted for 10.1% of those who reported having ever experienced a traumatic event (Table 1). Females had higher odds of lifetime PTSD compared to males in the total population (OR = 2.3). Females had higher odds of developing PTSD when exposed to a traumatic event (OR = 2.4). Females also had higher odds of experiencing current symptoms (OR = 2.4) in comparison to males.

**Table 1.** Lifetime and 12-month prevalence of DSM-IV post-traumatic stress disorder (PTSD).

	Lifetime								12 months			
	Total population				Trauma subsample				Total population			
	%	SE	*n*	Weighted *n*	%	SE	*n*	Weighted *n*	%	SE	*n*	Weighted *n*
Males	4.4	0.3	327	432,041	6.3	0.4	327	432,041	2.4	0.2	173	233,479
Females	9.7	0.5	808	974,005	13.7	0.6	808	974,005	5.6	0.3	475	563,778
Total	7.1	0.3	1135	1,406,045	10.1	0.4	1135	1,406,045	4.0	0.2	648	797,256

SE: standard error.

### Projected lifetime prevalence, age of onset, and remission of PTSD

It is projected that 9.6% of Australian adults will develop PTSD by the age of 85 (Table 2), with risk being greater for females in comparison to males (HR = 2.2, standard error [SE] = 0.1). The median age of onset of PTSD was 27 years, with 25% of people reporting onset by age 16% and 75% by age 47.

**Table 2.** Projected lifetime prevalence of DSM-IV post-traumatic stress disorder (PTSD) and age at selected age-of-onset percentiles.

	Projected lifetime prevalence at age 85 years		Age at selected age-of-onset percentiles (years)		
	%	SE	25	50	75
Males	6.4	0.6	17	30	51
Females	12.3	0.5	16	25	44
Total	9.6	0.4	16	27	47

SE: standard error.

The projected lifetime remission rate for PTSD was 63.3% overall, with males having a higher remission rate (68.7%) in comparison to females (61.2%, HR = 0.81; Table 3). Three quarters of this group entered remission after 10 years for males and 20 years for females. Analysis of projected cumulative remission showed that 18.0% of individuals with PTSD were expected to achieve symptom resolution within the first year (Table 4).

**Table 3.** Projected lifetime remission rates of post-traumatic stress disorder (PTSD) and projected length of time before entering remission.

	Projected remission rates				Projected length of time before entering remission (rounded years)		
	%	95% CI	HR	SE	25%	50%	75%
Males	68.7	[60.3, 75.3]	1	1	1	2	10
Females	61.2	[54.4, 66.9]	0.81	0.10	1	5	22
Total	63.3	[57.0, 68.7]			1	4	19

SE: standard error; HR: hazard ratio.

**Table 4.** Projected rates of remission for proportion of cohort with PTSD after set time periods.

	1 year		2 years		5 years		30 years	
	%	SE	%	SE	%	SE	%	SE
Males	21.5	2.33	29.0	2.6	39.2	1.49	57.3	3.38
Females	16.5	1.33	22.4	1.51	32.3	1.74	50.9	2.28
Total	18.0	1.16	24.3	1.31	33.2	1.49	52.7	1.89

SE: standard error.

### Comparison of 2020–2022 to 2007

There were no statistically significant differences in both lifetime and 12-month prevalence of PTSD between the 2007 and 2020–2022 surveys (Table 5). These patterns held across sex, with both males and females.

**Table 5.** Comparison of PTSD prevalence in 2020/2022 to 2007.

Lifetime PTSD							12-month PTSD					
2007			2020/2022				2007		2020/2022			
	% Yes	SE	% Yes	SE	OR	95% CI	% Yes	SE	% Yes	SE	OR	95% CI
Males	4.65	0.49	4.43	0.29	0.95	[0.73, 1.23]	2.78	0.35	2.39	0.23	0.86	[0.62, 1.18]
Females	9.75	0.57	9.68	0.46	0.99	[0.84, 1.17]	5.95	0.48	5.60	0.31	0.94	[0.76, 1.15]
Total	7.22	0.34	7.09	0.28	0.98	[0.86, 1.12]	4.38	0.26	4.02	0.21	0.92	[0.78, 1.08]

SE: standard error; OR: odds ratio; CI: confidence interval.

### Associations between PTSD, demographics, mental health, and physical health

Sociodemographic characteristics according to lifetime diagnosis of PTSD are presented in Table 6. Table 7 shows that having a lifetime diagnosis of PTSD was associated with a significantly higher odds of meeting diagnostic criteria for all other mental health disorders, suicidality, self-harm, and a range of physical health conditions, in comparison to not having a PTSD diagnosis. PTSD was temporally secondary for 65.7% of co-occurring mental disorders; however, temporal patterns differed according to disorder class (Table 8).

**Table 6.** Demographic characteristics stratified by lifetime post-traumatic stress disorder (PTSD) status.

	Lifetime PTSD			
	Yes	No	OR	95% CI
Age, mean (SE)	42.1 (0.6)	46.3 (0.1)	0.99	[0.98, 0.99]
Female, % (SE)	69.3 (1.6)	49.4 (0.1)	2.31	[1.96, 2.73]
Cisgender, % (SE)	98.4 (0.5)	99.7 (0.1)	0.19	[0.08, 0.43]
Heterosexual, % (SE)	88.4 (1.1)	95.5 (0.2)	0.36	[0.28, 0.45]
Married or de facto, % (SE)	38.3 (2.2)	52.3 (0.5)	0.57	[0.46, 0.69]
Born in Australia, % (SE)	77.8 (1.7)	65.8 (0.5)	1.83	[1.50, 2.23]
Major cities, % (SE)	75.5 (1.9)	76.1 (0.5)	0.97	[0.78, 1.20]
Completed year 12, % (SE)	77.0 (1.9)	78.1 (0.2)	0.94	[0.75, 1.18]
Completed non-school qualification, % (SE)	60.2 (2.1)	60.7 (0.3)	0.98	[0.81, 1.18]
Employed, % (SE)	65.6 (1.9)	65.4 (0.2)	1.01	[0.84, 1.20]
Pension, % (SE)	21.6 (1.5)	17.6 (0.4)	1.29	[1.07, 1.55]
Homeless, % (SE)	25.9 (1.7)	8.5 (0.3)	3.74	[3.11, 4.50]
Incarcerated, % (SE)	3.2 (0.7)	1.6 (0.1)	2.01	[1.24, 3.25]
Socioeconomic disadvantage, % (SE)	10.1 (1.3)	10.1 (0.3)	1.01	[0.75, 1.35]
Australia Defence Force, % (SE)	3.1 (0.7)	3.0 (0.2)	1.03	[0.65, 1.62]

Homeless: ever without a permanent place to live; Incarcerated: ever incarcerated; Socioeconomic 1st: index of relative socioeconomic disadvantage, percent of those in most disadvantaged (1st) quintile; Pension: government pensions and allowances are main source of income; Australian defence force: served in Australian defence force.

**Table 7.** Mental and physical health conditions according to lifetime diagnosis of post-traumatic stress disorder (PTSD).

**Mental health**						
	Lifetime PTSD					
	Yes		No			
	%	SE	%	SE	AOR	95% CI
**Anxiety disorders**						
Panic disorder	15.1	1.2	3.6	0.2	3.93	[3.15, 4.90]
Agoraphobia	16.1	1.6	2.9	0.2	5.60	[4.36, 7.19]
Social phobia	39.7	2.2	11.2	0.4	4.72	[3.89, 5.73]
Generalised anxiety disorder	32.0	1.9	8.4	0.3	4.47	[3.65, 5.48]
Obsessive compulsive disorder	17.8	1.5	4.5	0.2	4.07	[3.21, 5.15]
Any anxiety disorder, excluding PTSD	62.0	2.1	19.7	0.4	6.02	[4.99, 7.26]
**Mood disorders**						
Depressive episode	44.8	2.3	13.2	0.4	4.82	[3.90, 5.95]
Dysthymia	12.4	1.2	2.7	0.2	4.87	[3.67, 6.46]
Bipolar disorder	6.4	1.0	1.1	0.1	5.29	[3.51, 7.98]
Any mood disorder	45.6	2.3	13.7	0.4	4.77	[3.86, 5.90]
**Substance use disorders**						
Alcohol use disorder	33.1	1.8	16.0	0.4	3.24	[2.71, 3.87]
Drug use disorder	14.7	1.5	4.9	0.2	3.79	[2.88, 4.99]
Cannabis use disorder	11.6	1.3	4.0	0.2	3.58	[2.70, 4.74]
Amphetamine use disorder	6.2	0.9	2.3	0.2	3.10	[2.19, 4.39]
Opiate use disorder	2.0	0.6	0.4	0.1	5.41	[2.72, 10.76]
Sedative use disorder	3.4	1.0	0.5	0.1	7.86	[3.90, 15.88]
Any substance use disorder	37.0	1.8	17.4	0.4	3.45	[2.91, 4.08]
**Any disorder, excluding PTSD**	79.4	1.6	35.7	0.3	6.88	[5.60, 8.44]
**Suicide and self-harm**						
	Lifetime PTSD					
	Yes		No			
	%	SE	%	SE	AOR	95% CI
Thoughts	46.9	2.2	14.4	0.4	4.95	[4.15, 5.91]
Plans	27.0	2.2	6.0	0.3	5.46	[4.34, 6.87]
Attempts	17.7	1.4	3.9	0.2	4.87	[3.88, 6.12]
Any suicidal thoughts or behaviours	46.9	2.2	14.4	0.4	4.95	[4.15, 5.91]
Any self-harm	29.6	1.9	7.1	0.3	4.94	[3.94, 6.18]
**Physical health**						
	Lifetime PTSD					
	Yes		No			
	%	SE	%	SE	AOR	95% CI
Arthritis	19.9	1.7	17.4	0.4	1.64	[1.26, 2.13]
Osteoporosis	4.0	0.6	5.4	0.2	0.83	[0.60, 1.15]
Asthma	21.7	1.6	11.7	0.3	2.01	[1.67, 2.42]
Cancer	5.6	1.0	4.9	0.3	1.56	[0.98, 2.50]
Diabetes	8.5	1.3	6.7	0.3	1.76	[1.19, 2.61]
Heart disease	7.1	1.1	8.1	0.3	1.36	[0.93, 2.00]
Stroke	1.0	0.3	1.0	0.1	1.35	[0.68, 2.65]
Kidney disease	1.4	0.5	1.3	0.1	1.57	[0.72, 3.40]
Bronchitis or emphysema	4.6	0.7	3.1	0.2	1.87	[1.29, 2.70]
Other condition	25.5	1.5	17.5	0.4	1.71	[1.43, 2.05]
Any physical condition	71.1	1.9	51.2	0.4	2.92	[2.39, 3.58]

SE: standard error; AOR: adjusted odds ratio, controlled for age and sex; CI: confidence interval.

**Table 8.** Co-occurring disorders among those with lifetime post-traumatic stress disorder (PTSD).

	PTSD primary		PTSD same year		PTSD secondary	
	%	SE	%	SE	%	SE
Any anxiety disorder	23.4	2.0	11.4	1.4	65.3	2.3
Any mood disorder	35.5	2.7	22.6	2.8	41.9	3.1
Any substance use disorder	45.4	2.7	7.2	1.5	47.4	3.3
Any disorder^a^	21.0	1.7	13.4	1.6	65.7	2.1

SE: standard error.

a Any disorder: any anxiety (excluding PTSD), affective or substance use disorder.

## Discussion

This nationally representative analysis of the 2020–2022 NSMHW offers the most up-to-date estimates of PTSD burden and co-occurring mental and physical health conditions in Australia. The estimated prevalence of PTSD in Australia appears comparatively high when compared to international figures derived using similar WMH-CIDI and DMS-IV methodologies. Among high-income countries, the average lifetime prevalence is approximately 5% (Ferry et al., 2014; Iribarren et al., 2005; Kawakami et al., 2014; Koenen et al., 2017; Olaya et al., 2015), with even higher rates (>10%) observed among women and vulnerable groups, such as war veterans (Iribarren et al., 2005). Globally, the World Mental Health Surveys reported a pooled lifetime prevalence of 3.9% across 26 countries (Watson, 2019). Moreover, the conditional risk of PTSD, defined as the proportion of individuals with PTSD among those exposed to PTEs, was 10.1% in our study, exceeding the global average of 5.6% (Koenen et al., 2017) and the high-income country average of 6.9% (Watson, 2019). The higher prevalence observed in Australia relative to some other countries may reflect multiple factors. These may include genuine differences in patterns of trauma exposure, but may also arise from methodological differences in assessment and reporting practices that influence how traumatic experiences are recalled and endorsed in epidemiological surveys (Hardt and Rutter, 2004; Mills et al., 2011). Broader sociocultural and systemic factors may also influence how trauma-related experiences and symptoms are perceived, interpreted and reported across different populations (Chentsova-Dutton and Maercker, 2019; Ford et al., 2015). Cross-national comparisons are therefore subject to important measurement and contextual limitations and should be interpreted with caution.

The current study reaffirms the well-established finding that females are at greater risk of developing PTSD than males, with nearly double the lifetime prevalence observed in females compared to males. This pattern aligns with international data showing elevated PTSD risk among females and may reflect differences in trauma exposure type, biological vulnerability and social determinants of recovery (Darves-Bornoz et al., 2008; Luz et al., 2016). Finally, comparisons with the 2007 survey revealed no statistically significant change in prevalence over time, indicating a stable national burden over the past 15 years. The persistent prevalence underscores the ongoing need for effective prevention and intervention strategies.

The current study’s projected lifetime PTSD remission rate of 63.3% suggests that a substantial proportion will continue to experience persistent symptoms across the lifespan. This finding aligns with long-standing concerns about the chronicity of PTSD. For instance, earlier results from the U.S. National Comorbidity Survey found that approximately one-third of individuals with PTSD continued to meet diagnostic criteria after many years, even with treatment (Kessler et al., 1995). Meta-analyses have since estimated that nearly half of PTSD cases remain chronic (lasting more than 3 months after onset), with observed remission rates ranging widely between 8% and 89%, depending on trauma type, follow-up duration and sample characteristics (Morina et al., 2014; Steinert et al., 2015). The current study’s projected 12-month remission rate of 18% is lower than the 25–40% range reported in prior research, such as the World Mental Health Surveys (Koenen et al., 2017), highlighting potential variation in recovery trajectories across populations and study designs. These data reinforce evidence that for a subset of individuals, PTSD may follow a protracted or relapsing course, particularly in the absence of timely intervention (Steinert et al., 2015). Factors such as cumulative trauma exposure have been shown to significantly reduce the likelihood of remission, with higher traumatic load linked to more persistent symptoms (Kolassa et al., 2010).

Not only was the overall projected lifetime remission rate higher among males than females, but females also showed markedly slower recovery than males. Among those who achieved remission, half of males recovered within 2 years of symptom onset, compared with 4 years for females. Notably, one quarter of females remained symptomatic for over two decades. This prolonged symptom course among females may partly reflect differential trauma exposure, as females were more likely to experience interpersonal and intentional forms of trauma – such as domestic violence and sexual assault (Kroeger et al., 2025) – which are associated with a higher conditional risk and more chronic PTSD trajectories (Luz et al., 2016; Santiago et al., 2013). European data also point to elevated conditional risk for PTSD following events disproportionately experienced by women, including being raped, beaten by a partner or caregiver, and being stalked (Darves-Bornoz et al., 2008). Results from a nationally representative study in the United States indicate that although males and females had similar overall remission rates, the factors associated with remission differed by gender, suggesting that differences in time to remission may partly reflect distinct sociodemographic and clinical profiles (Stefanovics et al., 2022). Taken together, these findings support the growing recognition that sex differences in PTSD are shaped not only by biological susceptibility but also by the type, timing and context of trauma exposures. The persistence of symptoms among females underscores the importance of sex-responsive approaches to intervention and targeted research into mechanisms that sustain or disrupt recovery over time.

The 2020–2022 survey also reveals meaningful changes in PTSD remission patterns compared to the 2007 study (Chapman et al., 2012). In both surveys, short-term recovery trajectories were similar, with about one-third of affected individuals projected to recover within 5 years of symptom onset. This suggests relative stability in early remission over the past 15 years. However, the long-term outlook appears to have worsened. In 2007, the vast majority (more than nine-tenths) of individuals were expected to eventually recover, whereas the updated data project that only about one-half will recover, even decades after onset. This divergence may reflect changes in treatment access or update, evolving trauma exposure profiles, increased chronicity due to co-occurring conditions, or broader socioeconomic and environmental factors that hinder recover. One example of such a shift is that the odds of exposure to PTEs in the sexual assault category were higher in the 2020–2022 survey in comparison to 2007 (Kroeger et al., 2025). Although the reasons remain uncertain, the findings highlight the possibility that PTSD is becoming more enduring in the population, underscoring the importance of both timely intervention and sustained long-term support.

The demographic profile of individuals with PTSD in the 2020–2022 NSMHWB reveals a mixed pattern of findings. For several characteristics, including education level, employment status, area of residence, socioeconomic disadvantage and Australian Defence Force service, there were no statistically significant differences between those with and without PTSD. These results indicate that PTSD is not uniformly patterned across many commonly examined demographic domains. Among variables that did reach statistical significance, the magnitude of differences was sometimes modest. For example, individuals with PTSD were more likely to receive government pensions and to be born in Australia. However, the size of these effects was relatively small and may have limited practical implications at the population level. In contrast, more substantial differences were observed for specific indicators of social adversity. Individuals with PTSD were markedly more likely to have experienced homelessness and incarceration, and less likely to be married or in a de facto relationship. These findings are consistent with prior literature linking social marginalisation and instability to elevated risk and persistence of PTSD (Brewin et al., 2000, 2025; Kirkbride et al., 2024; Ozer et al., 2003). In addition, sexual and gender minority status was associated with increased odds of PTSD, consistent with meta-analytic evidence showing disproportionately high PTSD burden among transgender and gender-diverse populations (Marchi et al., 2023; Reisner et al., 2016). Overall, these findings suggest that while many demographic characteristics either show no difference or only small differences of limited practical importance, specific markers of social adversity and marginalisation remain strongly associated with PTSD burden.

The current findings reaffirm the substantial mental health burden associated with PTSD, consistent with prior international research. Individuals with a lifetime PTSD diagnosis in the 2020–2022 NSMHWB had markedly elevated odds of co-occurring anxiety, mood and substance use disorders, with co-occurrence present in most cases. These results align with earlier studies highlighting the high co-occurrence of PTSD with internalising and externalising disorders (Perkonigg et al., 2005; Shalev et al., 2017). Temporal sequencing analyses suggest that anxiety disorders precede the onset of PTSD nearly two-thirds of the time, highlighting their potential role in increasing risk following trauma exposure. However, this pattern may also reflect measurement-related factors, as pre-existing psychiatric conditions could influence how individuals perceive, appraise or report potentially traumatic events, thereby lowering the threshold at which experiences are classified as traumatic (Breslau et al., 2008; McLaughlin et al., 2010). In contrast, in this study, substance use disorders often emerge after PTSD onset or develop around the same time, which may reflect attempts to manage distress with substances (Jacobsen et al., 2001). Mood disorders also develop frequently in this study after PTSD diagnosis or during the same year. These patterns underscore the value of early detection and treatment of pre-existing anxiety, while also pointing to the need for integrated, trauma-informed approaches that address emerging substance use and mood disorders following PTSD diagnosis. The heightened risk of suicidality and self-harm – nearly half of those with PTSD reporting lifetime suicidal ideation – is also consistent with prior evidence linking chronic PTSD to heightened suicide risk (Tarrier and Gregg, 2004). These findings emphasise the complexity of PTSD presentations and the importance of early detection, coordinated care and suicide prevention efforts tailored to individuals with trauma histories.

The present study found that individuals with PTSD had significantly higher odds of reporting a range of physical health conditions, reinforcing the well-documented link between psychological trauma and somatic burden. Over 70% of those with PTSD reported at least one chronic physical condition, with particularly elevated rates of arthritis, diabetes and respiratory diseases, compared to about 50% of those without PTSD. These findings are consistent with extensive prior research highlighting PTSD’s co-occurrence with chronic physical conditions (Eaton, 2007; Miller and Sadeh, 2014; Shalev et al., 2017; Von Korff et al., 2009). Notably, asthma and bronchitis were nearly twice as common among those with PTSD in our sample, aligning with studies suggesting that altered inflammatory pathways and chronic stress–related dysregulation of the hypothalamic–pituitary–adrenal (HPA) axis may underlie respiratory vulnerability (Nilaweera et al., 2023). Similarly, the increased prevalence of diabetes and cardiovascular-related conditions is supported by evidence from large cohort and clinical studies indicating that PTSD contributes to atherosclerosis, metabolic dysfunction and reduced myocardial perfusion, often independent of traditional risk factors (Arenson and Cohen, 2017; Edmondson and von Kanel, 2017). These associations may be driven by both biological mechanisms – such as neuroendocrine and autonomic nervous system disruptions – and behavioural risk factors like substance use and sedentary lifestyle (van den Berk-Clark et al., 2018). These findings underscore the importance of integrated models of care that attend to both psychological and physical health in the aftermath of trauma.

Key strengths of this study include the large sample size and rigorous sampling methods, which enhance the generalisability of findings to the broader Australian adult population. Moreover, the study employed the WMH-CIDI, a structured and validated diagnostic instrument aligned with DSM-IV criteria, allowing for robust identification of PTSD and co-occurring disorders. In addition, the inclusion of projected lifetime prevalence and remission estimates offers valuable longitudinal insight despite the cross-sectional design, leveraging established statistical techniques to model long-term outcomes. The survey’s coverage of mental health, suicidality, self-harm, physical health conditions and key sociodemographic variables enables a multidimensional examination of PTSD burden and correlates.

Despite its methodological strengths, several limitations of the present study should be acknowledged. First, while the WMH-CIDI provides a validated framework for DSM-IV-based PTSD diagnosis, reliance on self-report and retrospective recall may introduce recall bias, particularly regarding age of onset, duration and exposure to traumatic events. As with all fully structured diagnostic interviews, the WMH-CIDI may present participants with a broader range of symptom presentations than would typically emerge in clinician-administered assessments, and responses may be influenced by expectations about how individuals believe they should respond after endorsing a traumatic event. Consequently, some cases identified through survey algorithms may meet DSM-IV criteria without necessarily reflecting clinically significant PTSD in a face-to-face assessment, potentially contributing to higher prevalence estimates. Conversely, prevalence estimates are also highly sensitive to variation in the type and number of traumas assessed, and underreporting of stigmatised experiences, such as sexual assault, may contribute to underestimates at the population level (Brewin et al., 2025). Differences between diagnostic systems also complicate comparisons with studies using other tools or criteria. For instance, the ICD-11 employs broader criteria for traumatic exposure, while some researchers advocate for symptom- and impairment-based definitions rather than rigid trauma qualifiers (Brewin et al., 2009; Gradus and Galea, 2022). Finally, because data were collected during the COVID-19 pandemic, 12-month prevalence estimates may have been influenced by pandemic-related stressors, service disruptions or changes in help-seeking and reporting. Although lifetime estimates are potentially less likely to be affected, the broader social context of the pandemic may have influenced responses and recall.

In summary, this study provides new nationally representative evidence on the prevalence, remission patterns, and associated burdens of PTSD in Australia. The observed sex differences in prevalence and remission underscore the importance of sex-informed approaches to prevention, early identification and long-term support for individuals with PTSD.
